# Assessing the Dynamics and Complexity of Disease Pathogenicity Using 4-Dimensional Immunological Data

**DOI:** 10.3389/fimmu.2019.01258

**Published:** 2019-06-12

**Authors:** Ariel L. Rivas, Almira L. Hoogesteijn, Athos Antoniades, Marios Tomazou, Tione Buranda, Douglas J. Perkins, Jeanne M. Fair, Ravi Durvasula, Folorunso O. Fasina, George P. Tegos, Marc H. V. van Regenmortel

**Affiliations:** ^1^School of Medicine, Center for Global Health-Division of Infectious Diseases, University of New Mexico, Albuquerque, NM, United States; ^2^Human Ecology, Centro de Investigación y de Estudios Avanzados (CINVESTAV), Mérida, Mexico; ^3^Stremble Ventures LTD, Limassol, Cyprus; ^4^Department of Pathology, School of Medicine, University of New Mexico, Albuquerque, NM, United States; ^5^Biosecurity and Public Health, Los Alamos National Laboratory, Los Alamos, NM, United States; ^6^Loyola University Medical Center, Chicago, IL, United States; ^7^Department of Veterinary Tropical Diseases, University of Pretoria, Pretoria, South Africa; ^8^Food and Agriculture Organization of the United Nations, Dar es Salaam, Tanzania; ^9^GAMA Therapeutics LLC, Mansfield, MA, United States; ^10^Centre National de la Recherche Scientifique (CNRS), School of Biotechnology, University of Strasbourg, Strasbourg, France

**Keywords:** personalized prognostics, pathogenesis, infection, inflammation, pattern recognition-based visualization

## Abstract

Investigating disease pathogenesis and personalized prognostics are major biomedical needs. Because patients sharing the same diagnosis can experience different outcomes, such as survival or death, physicians need new personalized tools, including those that rapidly differentiate several inflammatory phases. To address these topics, a pattern recognition-based method (PRM) that follows an inverse problem approach was designed to assess, in <10 min, eight concepts*: synergy, pleiotropy, complexity, dynamics, ambiguity, circularity, personalized outcomes*, and explanatory *prognostics* (pathogenesis). By creating thousands of secondary combinations derived from blood leukocyte data, the PRM measures synergic, pleiotropic, complex and dynamic data interactions, which provide personalized prognostics while some undesirable features—such as false results and the ambiguity associated with data circularity-are prevented. Here, this method is compared to Principal Component Analysis (PCA) and evaluated with data collected from hantavirus-infected humans and birds that appeared to be healthy. When human data were examined, the PRM predicted 96.9 % of all surviving patients while PCA did not distinguish outcomes. Demonstrating applications in personalized prognosis, eight PRM data structures sufficed to identify all but one of the survivors. Dynamic data patterns also distinguished survivors from non-survivors, as well as one subset of non-survivors, which exhibited chronic inflammation. When the PRM explored avian data, it differentiated immune profiles consistent with no, early, or late inflammation. Yet, PCA did not recognize patterns in avian data. Findings support the notion that immune responses, while variable, are rather deterministic: a low number of complex and dynamic data combinations may be enough to, rapidly, unmask conditions that are neither directly observable nor reliably forecasted.

## Introduction

Understanding the processes that, later, result in different outcomes–such as survival or death–, is an elusive goal. To improve research on disease pathogenesis, new methods are needed (1, 2), which should consider, at least, eight concepts: *synergy, pleiotropy, complexity, dynamics, ambiguity, circularity, personalized outcomes*, and *explanatory prognostics*.

Because living creatures are permanently interacting with the environment, they are both closed and open (3). While adjustments to changes originated in the environment may lead to higher costs, the costs imposed by higher complexity can be compensated with the lower costs induced by *synergic pleiotropy*, e.g., mutations in one gene can improve two or more traits without increasing the overall cost of complexity (4). Therefore, to assess living creatures that interact with their environment, new methods should measure both “one-to-many” (pleiotropic) and “many-to-one” (synergic) relationships, that is, “bow tie”-like processes (5).

The evidence that “one-to-many/many-to-one” constructs promote economically efficient biological systems is abundant: in spite of thousands of microbes potentially pathogenic, approximately 210 cell types suffice to confer survival (6). A few combinations of factors (cell types, in this example) perform many functions better, faster, and/or at a lower cost.

Consequently, the reductionist “one-to-one” (one structure/one function or “one target”) theory no longer holds (7). To capture pleiotropic synergies, new methods should measure two properties observed when living creatures respond to changes originated in their environment: *complexity* and *dynamic*s. In infectious diseases, complexity refers to the numerous combinations that host-microbial interactions may generate, which differ over time, i.e., they are dynamic (2).

Synergy, pleiotropy, complexity, and dynamics can express *ambiguity*: the same numerical value of the same variable does not always have the same meaning nor always performs the same function. Vice versa, different values of the same variable may be associated with the same function or meaning (8). For example, interleukin (IL) 6 is ambiguous. Given its pleiotropy–IL-6 is both a pro- and anti-inflammatory cytokine (9)–, its mere detection, in isolation and/or at a single time point, is error-prone. Similarly, monocytes are ambiguous: because they both promote neutrophil activity (at the beginning of an inflammation) and neutrophil destruction (at the end of the inflammation or recovery phase), measuring monocytes, alone and at a single time point, cannot distinguish between a new and a late inflammation (10).

Ambiguity may be characterized by *data circularity* (11). Because infections are temporal processes, they tend to express circular and ambiguous data. That is so because the functions performed by living creatures never stop. Consequently, no data pattern remains constant: if the individual survives, one pattern will eventually be replaced by a different (if not the opposite) pattern –a process that creates an oscillatory or circular shape. Thus, each value of each variable may, at least, have two meanings or perform two functions which are associated with either positive (feed forward) or negative feedback responses. For instance, similar values of mononuclear cells (MC or lymphocytes and monocytes) can predict both high and low MC/neutrophil (MC/N) ratios (8). The apparent ambiguity of the MC/N ratio may not be so but a valuable new piece of information: in the example mentioned above, septic patients that, after showing similar immunological values, exhibited opposite profiles, were infected by different bacteria, i.e., what seemed to be ambiguous could be distinguished (8). While circularity is observed when temporal data are plotted (11), tests conducted at a single time point may also display circularity or other non-linear patterns. Therefore, a group of individuals that experience different inflammatory phases may reveal distinct spatial patterns even when tested only once.

Randomization, alone, does not prevent ambiguity (11). However, 3D/4D *temporal data directionality* (arrows that denote where the data are coming from) may prevent ambiguity (8).

Because patients that share the same diagnosis may experience different outcomes, *prognosis*-oriented methods are needed (1, 12, 13). When based on personalized phenomena, prognostics may promote both research on disease pathogenesis and personalized practices. Such methods could detect immunomodulation before cytotoxicity occurs (1, 14).

In contrast, population-based models are unlikely to predict the outcome of a specific patient (15). Personalized prognostics may also avoid error-prone models. For example, classic statistics assume that the variables under analysis are *independently* distributed. However, immunological variables interact and, therefore, are *interdependen****t*** (2). That is the case of pleiotropic integrins (e.g., CD11b), which participate in many functions, including cell activation, transendothelial migration and phagocytosis (16, 17).

Hence, methods meant to prevent ambiguity can use numbers but should not depend on numerical assumptions. Pattern Recognition (PR) meets such criteria. Today facilitated by computerized technologies (e.g., “machine learning”), the spatial/temporal (4D) recognition of data patterns may apply to personalized medicine (18).

Biomedical methods can follow *reductionist* or *non-reductionist* theories. While, in the first group, omissions and/or errors are likely to occur because neither complexity nor dynamics are explored and dimensions are reduced (19), the second group does not reduce the number of dimensions and considers that biological systems are both complex and dynamic (2).

Methods may also be differentiated by the *problem* they investigate, which may be either *direct* (*forward* or *upstream*) or *inverse* (*downstream*). Inverse problem-based research starts with a result and then, following an inductive approach, goes back in history and looks for one or several possible cause(s). In contrast, direct problems start with known causes and, using deductive reasoning based on established mechanisms, collect new data to infer effects (20).

The *type of knowledge* generated can distinguish *invention-* from *discovery-*oriented methods (21). While the first type implements operations previously unfeasible, discovery-oriented methods unveil pre-existing but unknown phenomena (22). To our knowledge, invention- and discovery-oriented approaches have not yet been explored in Immunology.

*Metrics* can describe methods, too. Metrics can be something *directly measurable*, which is usually tested in isolation (e.g., counts or relative percentages of a given cell type) or *dimensionless* indices that capture *relationships* between two or more variables, such as the ratio between CD4+ and CD8+ lymphocyte counts or percentages (2, 23).

Methods also differ in the *question* they address. *Dichotomous* questions induce errors when three or more results are likely (11). For instance, methods that promote “yes/no” answers cannot assess inflammation, which can reveal three or more stages (no inflammation, early inflammation, late inflammation). Because they can offer *polychotomous* answers, pattern recognition-oriented methods prevent dichotomization-related errors (24).

Methods are also influenced by medical *needs*. For example, *real-time* information, as well as *data visualizations*, are needed (25, 26).

According to the *number of patients* under analysis, methods are cataloged as either *population*-oriented or *personalized* (27). When the number of patients is *n* = 1 (personalized medicine), no average (a population metric) can be produced and, therefore, no statistical analysis is possible. Methods that measure inverse problems and capture complex dynamics can be validated when they reveal a property typical of complex systems: *emergence* (28).

Emergence refers to patterns not directly observed when simple variables are measured in isolation, which become distinguishable when complex and dynamic interactions are analyzed (2). An emergence-based methodology grounds its reproducibility not on assumptions of unknown validity, but biologically explicit evidence.

Following an *inverse* problem approach, a non-reductionist, pattern recognition-oriented method (PRM) was developed to (a) capture *complex* and *dynamic interactions* (synergy and pleiotropy) that may express circularity; (b) prevent ambiguity; and (c) foster *personalized prognostics* (research on *pathogenesis*). This construct was evaluated with retrospective data collected from hantavirus-infected humans. To compare the informative ability of PRM, the same data were also explored with Principal Component Analysis or PCA (29). To estimate the reproducibility of the PRM, apparently healthy birds were also tested with both PRM and PCA.

Personalized prognosis is critical in hantavirus infections because its pathogenesis remains unclear and 20–40% of infected individuals may die within a couple of weeks (30). With two major clinical presentations (a hantavirus cardio-pulmonary [HCPS] and a hantavirus renal [HFRS] syndrome), efforts aimed at differentiating survival from non-survival are highly relevant in HCPS, where predictive factors have not yet been identified (31).

To estimate whether the PRM can capture functions conserved throughout evolution, avian data were also tested. Avian leukocyte data can inform on inflammation, a critical process in infectious and non-infectious diseases (32). For example, a documented inflammation may support diagnostics of septic infants (33). Because immunomodulation may occur in the absence of cytotoxicity, an unambiguous diagnosis of chronic inflammation is relevant in numerous fields, including toxicology, gerontology, and cancer (24, 34, 35).

These two datasets helped evaluate this proof-of-concept. Its purpose was to determine whether the PRM may extract more information than alternative methods.

## Materials and Methods

### Human Data

Following protocol numbers #13-463 and 16-084, a retrospective analysis investigated de-identified blood data collected from 40 humans (26 females and 14 males) admitted to the Health Science Center of the University of New Mexico (UNM), United States, where they were diagnosed as hantavirus-positive and treated, accordingly. These records included 8 fatalities.

### Avian Data

Avian blood samples (≤0.2 cc, *n* = 94) were collected or analyzed under protocol SGPA/SGVS/12648/13 of the Mexican Ministry for the Environment and Natural Resources. Clay colored thrushes (*Turdus grayi, n* = 72) and great-tailed grackles (*Quiscalus mexicanus, n* = 22) were sampled at: (i) an urban (20°58′47″N, 89°36′53″W) and (ii) a rural site (20°47′19″N, 89°35′26″W) of Mexico. The eosinophil counting method (Unopette Test 5877, Vacutainer Systems, BD Biosciences, Franklin Lakes, NJ, USA) was used to quantify white blood cells. Blood smears were stained with a modified Wright-Giemsa (Hematology Three-step Stain; Accra Lab, Bridgeport, NJ, USA). Differential cells counts were performed at the Environmental Health Laboratory of the Advanced Research Center (CINVESTAV), Merida, Yucatan, Mexico.

### Algorithm

Pattern recognition of blood data was facilitated by a proprietary algorithm (European Patent Office 2959295, 2018), which creates numerous data combinations among monocyte (M), neutrophil (N), and lymphocyte (L) counts or percentages. Its process is described in Supplementary Presentation SF 1–5). While all combinations are identical in primary (input) data, the number they generate (a dimensionless indicator or DI) differs for each combination. DIs –described here with a two- or three-letter identifier, e.g., *AAA*– are temporary guides used to recognize patterns. These acronyms do not refer to any known biological entity.

The algorithm consists of three steps, which (i) *create* and *expand* the number of complex data structures, (ii) keep only data structures that exhibit *distinct* patterns (data circularity, orthogonal data subsets, and data clusters), and (iii) *retrieve* the immune profile(s) associated with each data pattern. Steps I and II unmask hidden patterns. Step III identifies biologically interpretable indicators, removes artifacts, and releases new or unexpected information.

This procedure was conducted with blood leukocyte counts or relative percentages (Complete Blood Cell count or CBC). Each CBC of each patient was transformed into indices that included at least one interaction that involved at least two leukocyte cell types, which were then investigated as triplets in 3D/4D plots. A series of 3D analyses were then conducted, focusing each on: (i) temporal observations (if available), (ii) outcomes (if data on two or more outcomes were available), (iii) individuals, and/or (iv) pathogenesis (the immune profile specific of either survivors or non-survivors, or some feature of interest, e.g., immunomodulation). For comparison, PCA (a classic approach used in pattern recognition) was also utilized (36).

### Principal Component Analysis

Developed in 1901 (29), PCA both reduces the number of variables under analysis and explains most of the variance. When variables differ in scale, a correlation matrix is used, which gives equal weight to all variables. To that end, the data are standardized, i.e., the mean is subtracted. Thus, the standardized zero separates observations below the mean (negatives values) from those above the mean (positive values). Consequently, “positive” observations will be orthogonal and uncorrelated to “negative” ones. Otherwise, a covariance matrix is utilized (37).

### Validation

Four types of validity were assessed: (i) the ability to extract more information than alternatives (*construct* validity); (ii) the ability to convey similar information when different data structures are considered (*internal* validity); (iii) the ability to demonstrate similar findings when a different biological condition is tested in a different host, time, and/or place (*external* validity); and (iv) the reproducibility of a method when compared to a statistical alternative (*statistical* validity).

### Statistical Analysis

Medians were explored with the Mann-Whitney test (Minitab Inc, State College, PA, USA). The same software package was used to conduct PCA (including data standardization) and create 3D plots. To allow readers reproduce critical analyses, primary data, some of the complex variables derived from blood data, and related classes (e.g., survivor or non-survivor; no, early, or late inflammation) are shown in Supplementary Tables 1 and 2. Prognostic redundancy (i.e., whether two or more data structures assigned the same prognosis to the same person) is reported in Supplementary Table 3.

## Results

**Figure 1:**
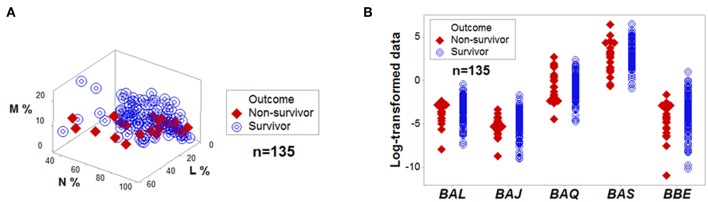
No variable, *per se*, is discriminant. Directly measured variables, such as the relative percentages of lymphocytes (L), monocytes (M), and neutrophils (N) did not separate outcomes of hantavirus-infected humans when measured in 3D space **(A)**. Complex and dimensionless variables (here identified as *BAL, BAJ, BAQ, BAS*, and *BBE*) also showed overlapping data distributions when survivors and non-survivors were measured **(B)**. The median L, M, and N percentages, as well as the median *BAL, BAJ, BAQ, BAS*, and *BBE* of survivors did not differ at a statistically significant level from those of non-survivors (*P* > 0.10, Mann-Whitney test).

**Figure 2:**
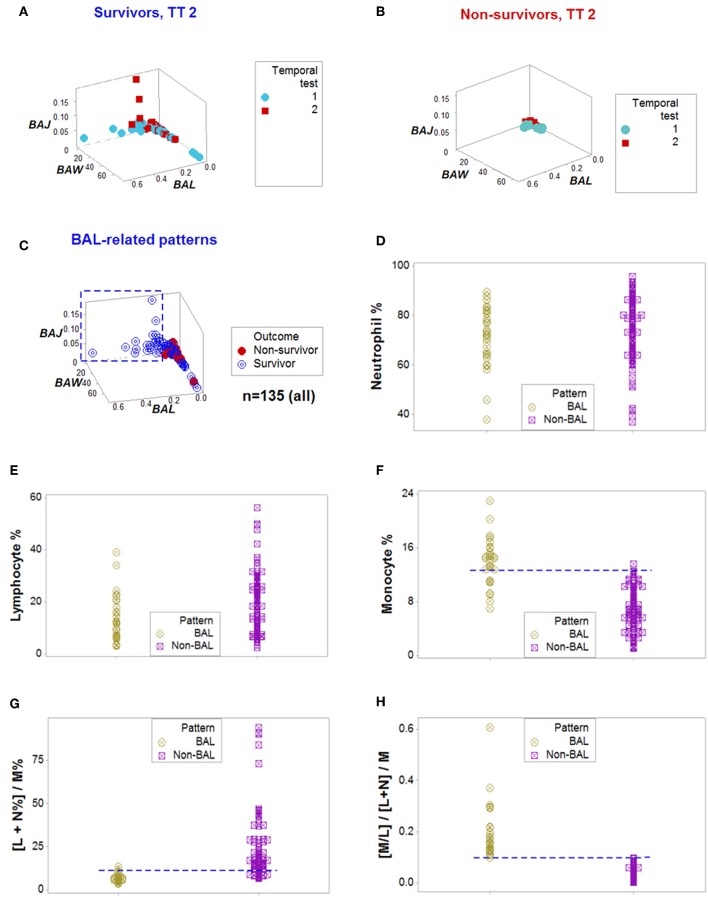
From pattern detection to biological validation. When spatial and temporal patterns were considered, as well as outcomes, emergent and non-random patterns were noticed since temporal test (TT) 2 **(A,B)**. While survivors displayed a perpendicular data departure at TT2 **(A)**, non-survivors exhibited observations with values ~0, at TT 1 and 2 **(B)**. The survivor-only pattern remained at all later testing times **(C)**. Personalized discrimination could be conducted even when the number of observations was *n* = 1: any one data point located with the rectangle shown in **(C)** could be predicted to survive. Validation of these patterns was based on a series of analyses of increasing complexity, which included biologically interpretable indicators **(D–H)**. While neither the neutrophil (N) nor the lymphocyte (L) percentages exhibited different intervals between the *BAL* and non-*BAL* data subsets **(D,E)**, some differences emerged whern the monocyte (M) percentage was assessed: a horizontal line, shows that most survivors captured by the *BAL* pattern displayed a higher M percentage, although a substantial data overlapping remained **(F)**. Because the sum of L % and N % is 100– M%, when both the L and N% are included in the numerator of a ratio that measures M% in the denominator, opposite relationships are expected in the two subsets analyzed, which were documented, although some data overlapping was still observed **(G)**. Replacing the M% with a relationship that includes two cell types (the M/L ratio), and also considering the ratio previously demonstrated to partially inform, totally discriminated the two (*BAL* and non-*BAL*) subsets **(H)**. Therefore, the subset that only included survivors–and was detectable since the second temporal test– was explained by a triple interaction, which included (i) the M/L and (ii) the [L+N]/M ratios, and (iii) the overall interaction that considered the two previous relationships.

Both percentages and complex indicators failed to separate the outcomes of hantavirus-infected patients (Figures 1A,B). Other indicators (including blood cell-related parameters, weight, body mass index, gender, and age) were also unable to distinguish survivors from non-survivors (Supplementary Presentation SF 6). Given the de-identified nature of the data analyzed, the influence of co-morbidities and prior conditions was not assessed. In spite of such limitations, several non-overlapping data distributions differentiated survivors from non-survivors when complex indicators were measured in 3D space and time, as well as outcomes, were also considered (Figures 2A–C and Supplementary Video 1). Analyses conducted with immunological indicators of increasing complexity validated such findings (Figures 2D–H).

**Figure 3:**
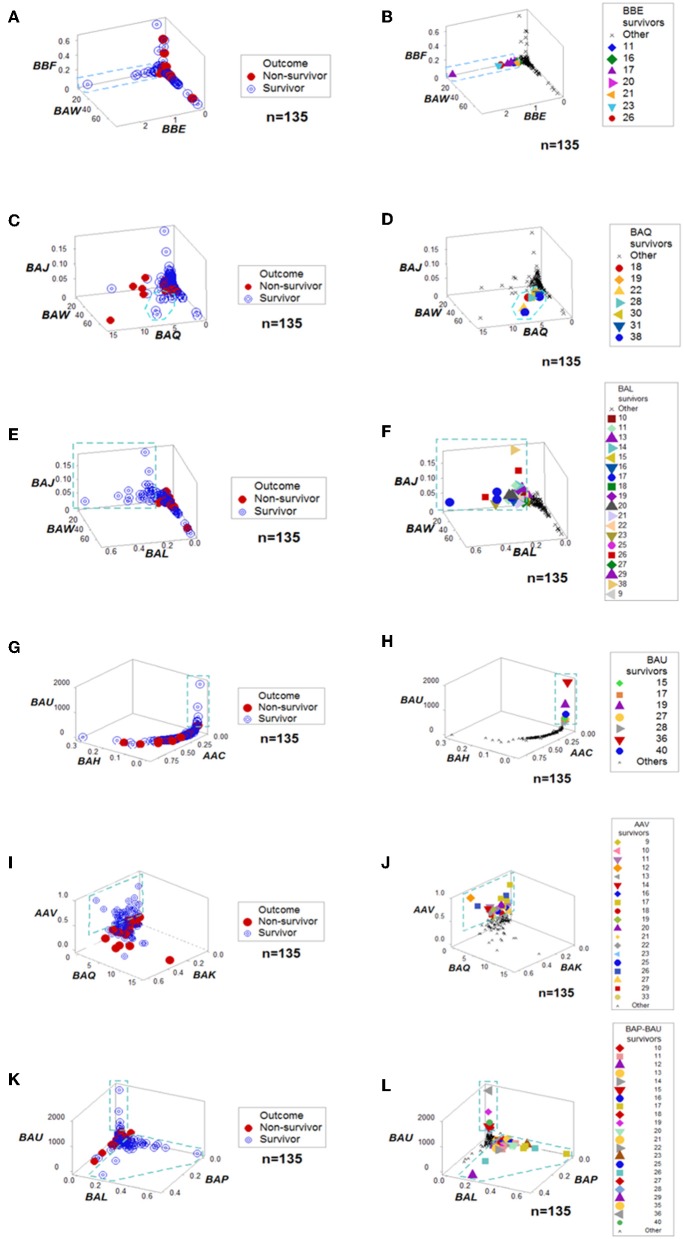
Outcome-specific discrimination. **Six** data structures revealed distinct patterns among surviving hantavirus-infected humans **(A–L)**. Redundancy (detection of a similar finding, when a different data structure is considered) was demonstrated. For instance, patient #11 was identified as a “survivor” by four data structures **(B,F,J,L)**. Numbers refer to patient identifiers. “Other” refers to both other survivors and all non-survivors.

**Figure 4:**
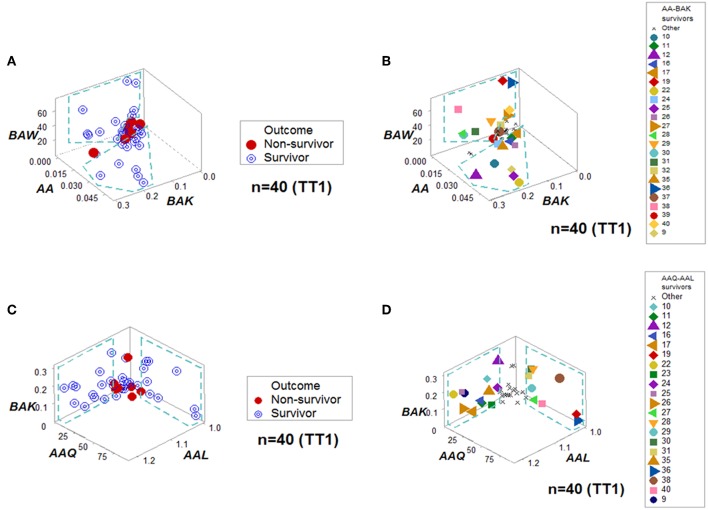
Personalized prediction for survival at admission. Two data structures clustered, at the first temporal test (TT1), 75% (24/32) of all survivors within data ranges that did not include non-survivors. **(A,B)** denote the patterns and patient identifiers of the first data structure, while **(C,D)** display those of the second data structure. Twenty of the twenty-four survivors (83.3%) were redundantly predicted.

When all the longitudinal data were examined, six data structures predicted 87.5% of all survivors (Figures 3A-L). Outcomes were not randomly distributed: several data structures showed spatially distinct subsets –e.g., orthogonal inflections of data points–, which were only composed of survivors (Figures 3A,C,E,G,I,K). Therefore, discrimination was data-, not hypothesis-driven. While human patients may experience different disease stages at the time they are hospitalized, two additional data structures that only included data available at the first temporal test identified 75% of all survivors (Figures 4A–D). When eight data structures were considered –two that measured the first temporal test and six data structures that considered all temporal data points– 96.9% (31/32) of all survivors were identified (Figures 3, 4, and ST3).

**Figure 5:**
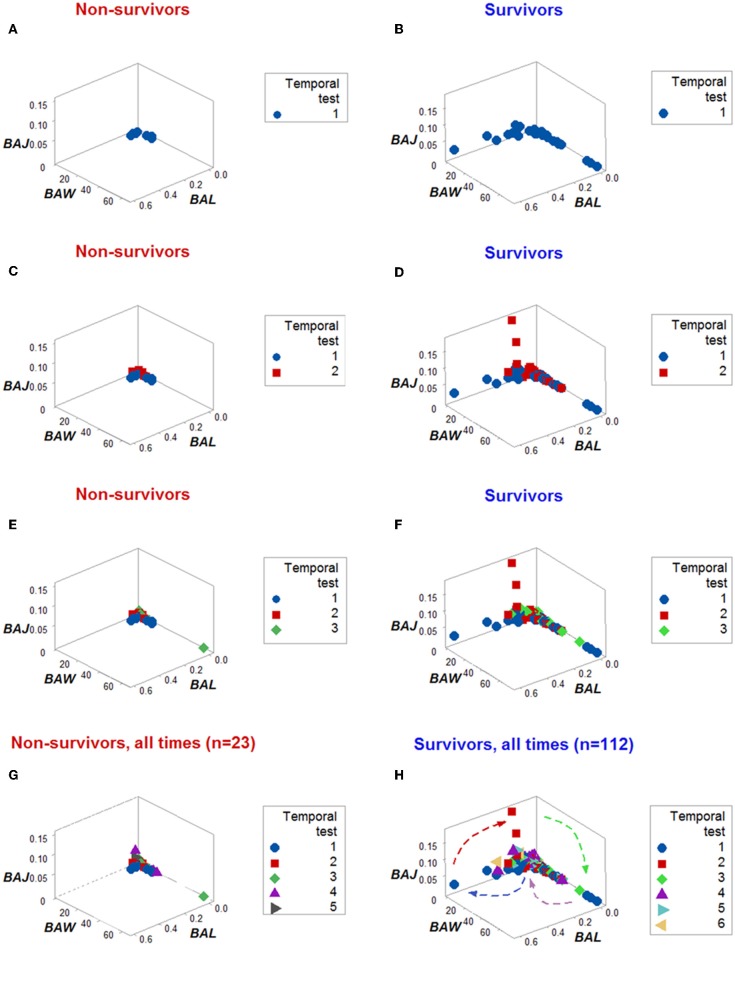
Dynamic pattern-related prognostics (I): survivors. When only the temporal order –not the actual dates– in which patients were tested was plotted, the spatial-temporal patterns created by the directionality of the data distinguished non-survivors **(A,C,E,G)** from survivors **(B,D,F,H)**.

**Figure 6:**
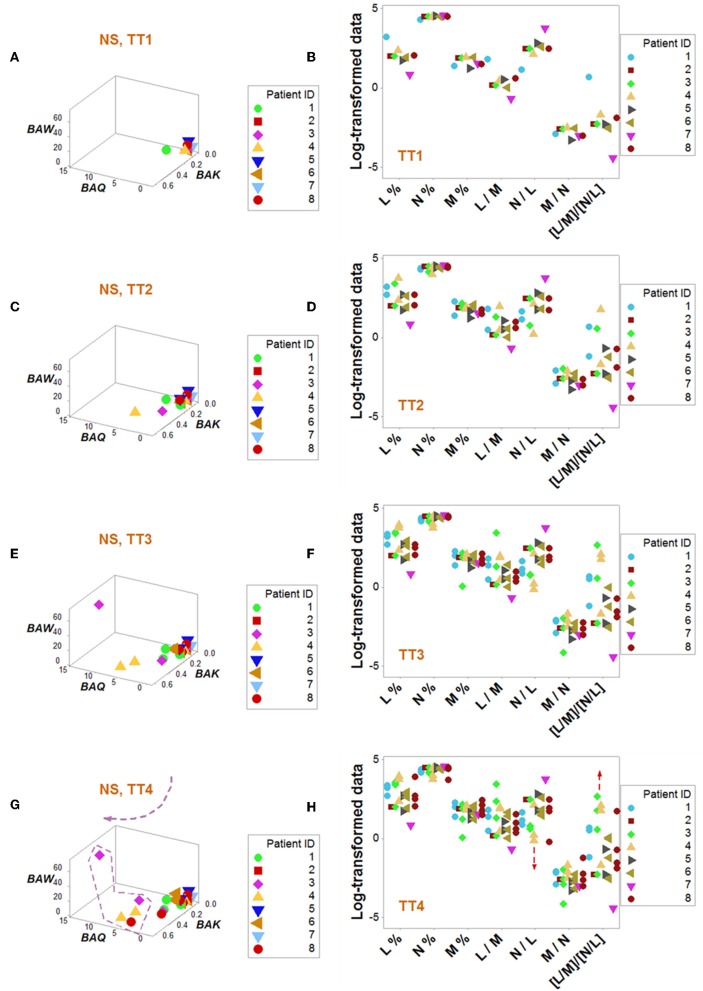
Dynamic pattern-related prognostics (II): non-survivors. Spatial-temporal patterns of non-survivors revealed two subsets **(A–H)**: a subset detected at later times showed lower N/L and higher [L/M]/[N/L] values, i.e., a chronic inflammation **(G,H)**.

**Figure 7:**
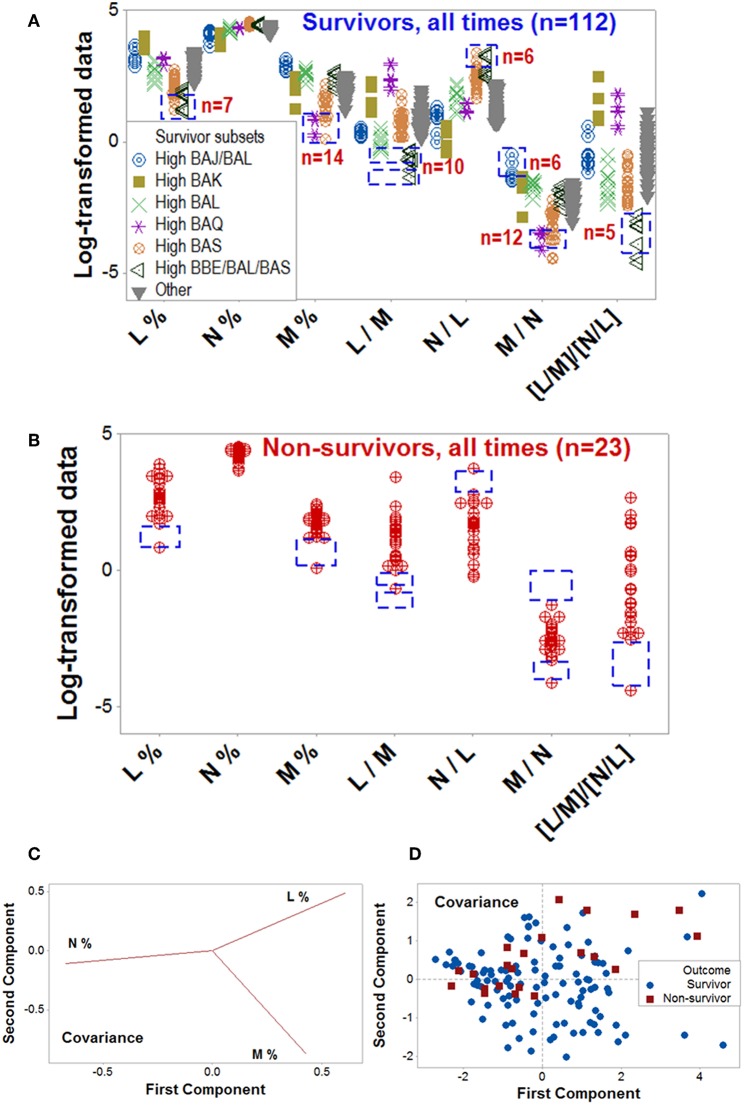
Validation of data patterns and assessment of pathogenesis. The data ranges of some biologically interpretable variables or relationships were explored with data collected at all time points, from all patients (*n* = 135). Supplementary Video 1 shows a temporal assessment of pathogenesis. Rectangles show some data ranges contributed by survivors **(A)**, which were not displayed by non-survivors **(B)**. While the number of survivors (*n* = 32) was 4 times higher than non-survivors (*n* = 8), some differences exceeded the 4:1 ratio, e.g., survivors reported 10 observations within a monocyte percentage interval within which no non-survivor contributed any data point (rectangles, **A,B**). Unlike the PRM, PCA did not discriminate hantavirus-related outcomes: while the loading plots displayed distinct differences between the three input variables **(C)**, such differences did not result in pattern recognition **(D)**. Because the PCA conducted with the covariance alternative yielded similar results, it is not shown. Initials are defined in Figure 1.

Dynamics differentiated outcomes. Temporal data inflections (directionalities revealed by arrows that connected temporal points) demonstrated, in survivors, at least four data patterns that non-survivors did not reveal (Figures 5A–H). Spatial dynamics also identified one non-survivor subset that, later, expressed chronic inflammation (higher [L/M]/[N/L] values; Figures 6A–H). Spatial-temporal data patterns also revealed immunological differences. For instance, the high *BAL* subset displayed a lower and non-overlapping interval of L percentages compared to the high *BAK* subset (Figure 7A). Validations also informed on pathogenesis: data ranges of immune functions found in survivors were not observed in non-survivors (rectangles, Figures 7A,B). These differences were not due to the 4 times larger number of survivors (*n* = 32) than non-survivors (*n* = 8): several data ranges expressed by survivors included more than 4 observations (Figure 7A). In contrast, PCA did not separate hantavirus-related outcomes (Figures 7C,D).

**Figure 8:**
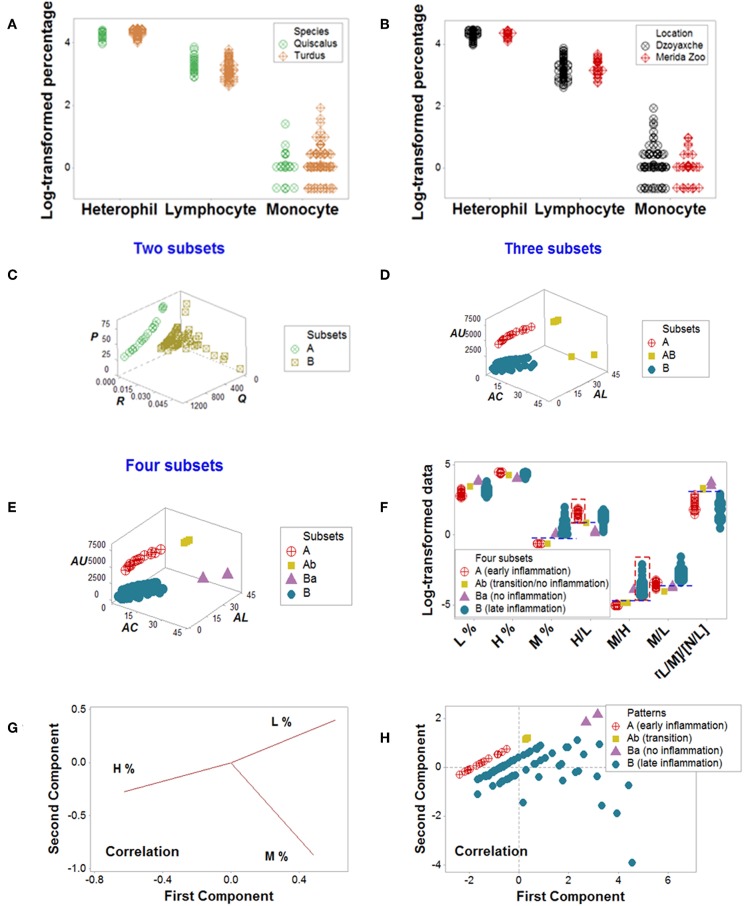
Avian data—PRM and PCA assessments. Simple blood leukocyte indicators did not distinguish geographical sites or avian species **(A,B)**. Several complex data structures revealed the presence of up to four subsets **(C–E)**. When analyzed with biologically interpretable indicators, the data subsets displayed patterns consistent with no, early, or late inflammation **(F)**. The late inflammation group (subset **B**) showed a significantly higher median M% than the early inflammation group (subset **A**, *P* < 0.01). Subset B exhibited a significantly higher median M/H than subset A (*P*<0.01, **F**). In contrast, PCA did not discriminate **(G, H)**. Initials: lymphocytes (L), monocytes (M), and heterophils (H, the avian counterpart of mammalian neutrophils).

Cross-sectional avian data revealed similar information: classic analyses did not distinguish patterns, even when avian species and geographical location were controlled for (Figures 8A,B). Yet, four data patterns were differentiated when complex indicators were explored in 3D space, which showed non-overlapping intervals of immunologically interpretable variables (Figures 8C–E). While the PRM identified immune profiles compatible with no, early, or late inflammation (Figure 8F), PCA did not reveal patterns (Figures 8G,H).

## Discussion

### Overview

While the ability to predict mortality in other disease states has been as high as 86% (38–41), the PRM identified 96.9% of survivors. The similar ranges of neutrophil data found in survivors and non-survivors (Figures 5A,B), together with the mononuclear cell-related data differences displayed across outcomes are compatible with a monocyte-mediated increased endothelial permeability previously reported in hantavirus-infected patients (42, 43). As expected, more information was extracted from structured than non-structured data (9, 44–47). A deterministic process was suggested by the fact that eight data structures were enough to prognosticate all but one of the infected survivors. Such a low number of data structures ruled out the hypothesis of random interactions –which would result in a quasi-infinite number of combinations (28)– and also facilitated a rapid analysis.

Emergent immunological patterns were both informative and explanatory. In humans and birds, the PRM identified chronic inflammation. In agreement with reports that describe human persistent inflammation is associated with late (≥14 days) in-hospital death (48), a subset of non-survivors exhibited higher [L/M]/[N/L] values at or after the third temporal test (Figures 6A–H). Birds also displayed a subset characterized by statistically significantly higher median blood monocyte percentages than those characterized by no or early inflammation (Figure 8F). While the mere detection of late inflammation is not a cause of concern, it should be explained when it persists, i.e., when late inflammation becomes chronic (34, 35).

While only cellular variables were investigated in this proof-of-concept, earlier studies have shown that other biological scales –e.g., cell surface molecules– can also be assessed with this method (45, 46). Thus, this multi-scale method can both reveal “one-to-many” and “many-to-one” interactions (“inventions”) and perform validations that unmask (“discover”) complexity (2).

### Discoveries

To both visualize underlying patterns and prevent false results, complexity was artificially created and, then, validated. Following established computational approaches (49), the data were artificially augmented with complex indicators. The *temporary use of artifacts* is a strategy reported in the History of Science: Euclid developed Geometry using a definition (a line is a length or distance *lacking thickness*) known to be false, although operationally useful (50). To reveal functions that operate over different temporal scales, complex dynamics were not measured with chronological scales, but with biological concepts (e.g., individualized history, complex interactions, and discrete outcomes). To prevent errors, three strategies were applied: (i) pattern recognition, (ii) noise reduction, and (iii) redundancy.

Recognition of immunological patterns occurs when indicators are designed to show some features, such as those of ‘anchors’ and/or ‘amplifiers’ (11). *Pattern recognition* is further fostered when large numbers of data combinations are derived from the primary (directly measurable) data (45, 47). *Noise reduction* is one special case of pattern recognition, in which one data point-wide lines of observations are created (44). Because no data variability exists in one data point-wide lines (except along the line), noise is reduced, discrimination is enhanced, temporal changes will be detected because they can only occur along the line, and –when ratios are used– they will be noticed earlier than when counts or percentages are evaluated (44).

“Discoveries” were induced when outcomes were measured and redundancy was practiced (28). To “discover”, neither conceptualizations were essential nor hypothesis-driven research was required. Instead, inferences were based on perception-centered learning, such as data visualizations (51–53). Findings supported the view that evolution is not a random process (54): eight data combinations sufficed to identify all but one of the hantavirus-infected survivors.

### The Invention/Discovery Connection

To promote “discoveries” that change over time, two “inventions” were needed. The first “invention” was the artificial complexity introduced in the first step of the analysis. Yet, the PRM construct, alone, did not foster “discoveries” (Figure 1B). Pre-existing but previously unknown phenomena –e.g., information on pathogenesis– were only released when >1000 data combinations were plotted with a spatial and temporal (4D) format. The second “invention” was a movie–like presentation. Only the composite (double invention- and discovery-oriented) method provided a combinatorial and yet, concise operation, which solved numerous problems, including the need for *rapid* analyses. The combination of hundreds of dynamic plots and a few static figures exposed cloistered patterns, providing a visual process that uses blood leukocytes and concludes within minutes (Supplementary Video 1).

### Similarities, Differences, and Complementation With Alternative Approaches

While both PRM and PCA aim at discovering occult patterns, their strategy differs: while PCA emphasizes dimension *reduction*, the PRM *increases* the number of data structures available for analysis (2). The inability of PCA to discriminate was not unexpected: PCA is sensitive to linearity and sample size and, in addition, is not well-suited to capture dynamics (36, 55, 56).

It is suggested that the invention- and discovery-oriented method could facilitate the first step (“unsupervised” learning) of machine learning (57). The movie-like features of the PRM could also circumvent the central limitation of printed formats: static information (58).

### A Three-In-One Approach

The expanded assessment of biological systems could start a new testing paradigm. If, in addition to the few variables analyzed here, the geo-referenced location of the host and genomic information on the pathogen were also recorded, the three dimensions that involve infections could be simultaneously assessed: the host, the pathogen, and the environment. By capturing many environmental scales –ranging from multi-cellularity (as shown here) to biogeography (59)–, such a method could (i) prognosticate outcomes; (ii) explore pathogenesis; (iii) detect super-spreading pathogens; and (iv) control epidemics (60–62).

## Future Steps

Inverse problems were examined with several (although not too many) data structures, which assessed well-conserved properties of biological systems (28, 63, 64). While direct or forward problems have historically predominated in Biomedicine, when the goal is to “discover” what, usually, is not observable, inverse problem-oriented methods may yield more information even when the size of the data analyzed is small–as demonstrated here. Thus, “discovery”-oriented methods may offer an alternative to approaches that require very large datasets, such as “deep learning” (65). It is suggested that, to fully validate, prospective data and interdisciplinary collaborations that include clinicians and methodologists are required.

## Author Contributions

AR, GT, and MvR conceived the study. AA, AH, FF, MT, and TB contributed materials, reagents, and data. AR, DP, MvR, JF, and RD wrote the paper.

### Conflict of Interest Statement

AR and AH are co-inventors of the temporary guides used to recognize patterns (European Patent Office 2959295). AA and MT were employed by company Stremble Ventures LTD. GT was employed by company GAMA LLC. The remaining authors declare that the research was conducted in the absence of any commercial or financial relationships that could be construed as a potential conflict of interest.
