# Supplementary material for: Assessing the Dynamics and Complexity of Disease Pathogenicity Using 4-Dimensional Immunological Data
Source: Front Immunol. 2019 Jun 12;10:1258. doi: 10.3389/fimmu.2019.01258 (PMC6582751; doi:10.3389/fimmu.2019.01258)
Supplement: Supplementary file 1 [file Table_1.DOCX]

SUPPLEMENTARY MATERIAL

Table S1. Hantavirus-related data.

| **Patient ID** | **Outcome** | **TT*** | **L %*** | **N %*** | **M %*** | ***BAJ**** | ***BAL**** | ***BAW**** |
| --- | --- | --- | --- | --- | --- | --- | --- | --- |
| 1 | Non-survivor | 1 | 23.68 | 72.36 | 3.94 | 0.0022 | 0.0068 | 15.71 |
| 2 | Non-survivor | 1 | 7.36 | 86.31 | 6.31 | 0.0049 | 0.0577 | 7.35 |
| 3 | Non-survivor | 1 | 7.36 | 86.31 | 6.31 | 0.0049 | 0.0577 | 7.35 |
| 4 | Non-survivor | 1 | 10.11 | 83.14 | 6.74 | 0.0058 | 0.0481 | 7.40 |
| 5 | Non-survivor | 1 | 5.55 | 91.11 | 3.33 | 0.0012 | 0.0206 | 17.08 |
| 6 | Non-survivor | 1 | 6.74 | 86.51 | 6.74 | 0.0056 | 0.0722 | 6.41 |
| 7 | Non-survivor | 1 | 2.24 | 93.25 | 4.49 | 0.0022 | 0.0941 | 6.91 |
| 8 | Non-survivor | 1 | 7.52 | 88.17 | 4.30 | 0.0021 | 0.0256 | 13.04 |
| 9 | Survivor | 1 | 14.47 | 72.36 | 13.15 | 0.0275 | 0.1377 | 2.88 |
| 10 | Survivor | 1 | 18.08 | 73.40 | 8.51 | 0.0107 | 0.0437 | 5.86 |
| 11 | Survivor | 1 | 6.02 | 83.13 | 10.84 | 0.0158 | 0.2189 | 2.73 |
| 12 | Survivor | 1 | 27.05 | 67.05 | 5.88 | 0.0054 | 0.0135 | 9.36 |
| 13 | Survivor | 1 | 10.41 | 84.37 | 5.20 | 0.0033 | 0.0274 | 10.80 |
| 14 | Survivor | 1 | 7.14 | 86.90 | 5.95 | 0.0043 | 0.0527 | 7.96 |
| 15 | Survivor | 1 | 6.74 | 87.64 | 5.61 | 0.0038 | 0.0496 | 8.50 |
| 16 | Survivor | 1 | 7.31 | 81.70 | 10.97 | 0.0165 | 0.1849 | 2.97 |
| 17 | Survivor | 1 | 3.29 | 83.51 | 13.18 | 0.0239 | 0.6075 | 1.26 |
| 18 | Survivor | 1 | 10.00 | 85.55 | 4.44 | 0.0024 | 0.0206 | 13.32 |
| 19 | Survivor | 1 | 6.52 | 92.39 | 1.08 | 0.0001 | 0.0018 | 72.85 |
| 20 | Survivor | 1 | 23.15 | 72.63 | 4.21 | 0.0025 | 0.0079 | 14.59 |
| 21 | Survivor | 1 | 3.48 | 89.53 | 6.97 | 0.0058 | 0.1500 | 4.27 |
| 22 | Survivor | 1 | 16.04 | 69.13 | 14.81 | 0.0372 | 0.1605 | 2.42 |
| 23 | Survivor | 1 | 3.06 | 87.75 | 9.18 | 0.0105 | 0.3033 | 2.38 |
| 24 | Survivor | 1 | 12.04 | 80.72 | 7.22 | 0.0069 | 0.0467 | 6.97 |
| 25 | Survivor | 1 | 15.85 | 70.73 | 13.41 | 0.0293 | 0.1310 | 2.85 |
| 26 | Survivor | 1 | 6.74 | 78.65 | 14.60 | 0.0317 | 0.3706 | 1.70 |
| 27 | Survivor | 1 | 5.19 | 92.20 | 2.59 | 0.0007 | 0.0133 | 23.66 |
| 28 | Survivor | 1 | 23.07 | 74.72 | 2.19 | 0.0006 | 0.0021 | 31.04 |
| 29 | Survivor | 1 | 11.68 | 85.71 | 2.59 | 0.0008 | 0.0059 | 27.00 |
| 30 | Survivor | 1 | 22.98 | 74.71 | 2.29 | 0.0007 | 0.0023 | 29.54 |
| 31 | Survivor | 1 | 18.42 | 78.94 | 2.63 | 0.0009 | 0.0038 | 26.25 |
| 32 | Survivor | 1 | 8.33 | 88.09 | 3.57 | 0.0015 | 0.0158 | 17.26 |
| 33 | Survivor | 1 | 9.30 | 83.72 | 6.97 | 0.0062 | 0.0562 | 6.85 |
| 34 | Survivor | 1 | 6.38 | 88.29 | 5.31 | 0.0033 | 0.0468 | 9.05 |
| 35 | Survivor | 1 | 11.11 | 80.00 | 8.88 | 0.0108 | 0.0780 | 5.00 |
| 36 | Survivor | 1 | 3.29 | 95.60 | 1.09 | 0.0001 | 0.0037 | 65.25 |
| 37 | Survivor | 1 | 9.37 | 85.93 | 4.68 | 0.0026 | 0.0245 | 12.22 |
| 38 | Survivor | 1 | 23.52 | 75.29 | 1.17 | 0.0001 | 0.0005 | 60.95 |
| 39 | Survivor | 1 | 12.63 | 82.10 | 5.26 | 0.0035 | 0.0231 | 11.01 |
| 40 | Survivor | 1 | 4.30 | 93.54 | 2.15 | 0.0005 | 0.0109 | 29.00 |
| 1 | Non-survivor | 2 | 14.66 | 76.00 | 9.33 | 0.0126 | 0.0655 | 4.97 |
| 3 | Non-survivor | 2 | 29.76 | 61.90 | 8.33 | 0.0122 | 0.0254 | 5.80 |
| 4 | Non-survivor | 2 | 42.16 | 51.80 | 6.02 | 0.0074 | 0.0091 | 7.52 |
| 5 | Non-survivor | 2 | 14.28 | 80.61 | 5.10 | 0.0034 | 0.0192 | 11.64 |

**Table S1. (cont’d)**

| **Patient ID** | **Outcome** | **TT** | **L %** | | **N %** | **M %** | ***BAJ*** | ***BAL*** | ***BAW*** |
| --- | --- | --- | --- | --- | --- | --- | --- | --- | --- |
| 6 | Non-survivor | 2 | | 13.33 | 78.88 | 7.77 | 0.0083 | 0.0491 | 6.40 |
| 8 | Non-survivor | 2 | | 14.60 | 79.77 | 5.61 | 0.0041 | 0.0228 | 10.25 |
| 9 | Survivor | 2 | 24.74 | | 63.91 | 11.34 | 0.0226 | 0.0586 | 3.86 |
| 10 | Survivor | 2 | 25.51 | | 64.28 | 10.20 | 0.0180 | 0.0454 | 4.50 |
| 11 | Survivor | 2 | 17.72 | | 67.08 | 15.18 | 0.0405 | 0.1535 | 2.37 |
| 12 | Survivor | 2 | 39.08 | | 37.93 | 22.98 | 0.1809 | 0.1755 | 1.03 |
| 13 | Survivor | 2 | 16.16 | | 73.73 | 10.10 | 0.0153 | 0.0702 | 4.49 |
| 14 | Survivor | 2 | 27.71 | | 60.24 | 12.04 | 0.0273 | 0.0595 | 3.48 |
| 15 | Survivor | 2 | 9.00 | | 82.00 | 9.00 | 0.0108 | 0.0989 | 4.55 |
| 17 | Survivor | 2 | 8.98 | | 85.39 | 5.61 | 0.0039 | 0.0372 | 9.35 |
| 18 | Survivor | 2 | 22.82 | | 69.56 | 7.60 | 0.0090 | 0.0274 | 6.85 |
| 19 | Survivor | 2 | 20.21 | | 63.82 | 15.95 | 0.0474 | 0.1499 | 2.23 |
| 20 | Survivor | 2 | 11.34 | | 74.22 | 14.43 | 0.0327 | 0.2146 | 2.26 |
| 21 | Survivor | 2 | 19.19 | | 74.74 | 6.06 | 0.0052 | 0.0203 | 9.37 |
| 22 | Survivor | 2 | 15.00 | | 78.75 | 6.25 | 0.0052 | 0.0277 | 8.89 |
| 24 | Survivor | 2 | 7.29 | | 87.50 | 5.20 | 0.0032 | 0.0392 | 9.80 |
| 26 | Survivor | 2 | 34.04 | | 45.74 | 20.21 | 0.1119 | 0.1504 | 1.42 |
| 27 | Survivor | 2 | 5.74 | | 86.20 | 8.04 | 0.0081 | 0.1225 | 4.46 |
| 28 | Survivor | 2 | 6.41 | | 91.02 | 2.56 | 0.0007 | 0.0105 | 25.35 |
| 29 | Survivor | 2 | 24.32 | | 70.27 | 5.40 | 0.0043 | 0.0126 | 10.63 |
| 30 | Survivor | 2 | 5.81 | | 90.69 | 3.48 | 0.0013 | 0.0216 | 16.25 |
| 31 | Survivor | 2 | 22.07 | | 66.23 | 11.68 | 0.0233 | 0.0700 | 3.70 |
| 33 | Survivor | 2 | 16.12 | | 75.26 | 8.60 | 0.0107 | 0.0501 | 5.70 |
| 34 | Survivor | 2 | 10.46 | | 81.39 | 8.13 | 0.0088 | 0.0689 | 5.62 |
| 36 | Survivor | 2 | 7.86 | | 88.76 | 3.37 | 0.0013 | 0.0149 | 18.43 |
| 37 | Survivor | 2 | 24.48 | | 66.32 | 9.18 | 0.0140 | 0.0379 | 5.25 |
| 38 | Survivor | 2 | 18.18 | | 79.54 | 2.27 | 0.0006 | 0.0029 | 31.11 |
| 39 | Survivor | 2 | 15.55 | | 78.88 | 5.55 | 0.0041 | 0.0210 | 10.46 |
| 1 | Non-survivor | 3 | 28.04 | | 64.63 | 7.31 | 0.0089 | 0.0205 | 7.00 |
| 3 | Non-survivor | 3 | 31.57 | | 67.36 | 1.05 | 0.0001 | 0.0003 | 61.93 |
| 4 | Non-survivor | 3 | 50.00 | | 42.30 | 7.69 | 0.0151 | 0.0128 | 4.76 |
| 6 | Non-survivor | 3 | 18.60 | | 76.74 | 4.65 | 0.0029 | 0.0121 | 13.20 |
| 8 | Non-survivor | 3 | 11.76 | | 80.00 | 8.23 | 0.0092 | 0.0628 | 5.71 |
| 10 | Survivor | 3 | 20.68 | | 64.36 | 14.94 | 0.0407 | 0.1268 | 2.50 |
| 11 | Survivor | 3 | 25.77 | | 62.88 | 11.34 | 0.0230 | 0.0562 | 3.85 |
| 13 | Survivor | 3 | 20.61 | | 67.01 | 12.37 | 0.0260 | 0.0847 | 3.38 |
| 14 | Survivor | 3 | 29.16 | | 66.66 | 4.16 | 0.0027 | 0.0062 | 14.00 |
| 15 | Survivor | 3 | 12.76 | | 80.85 | 6.38 | 0.0053 | 0.0340 | 8.44 |
| 17 | Survivor | 3 | 6.38 | | 91.48 | 2.12 | 0.0005 | 0.0072 | 32.25 |
| 18 | Survivor | 3 | 33.72 | | 62.79 | 3.48 | 0.0020 | 0.0037 | 16.31 |
| 19 | Survivor | 3 | 21.34 | | 75.28 | 3.37 | 0.0015 | 0.0055 | 19.28 |
| 20 | Survivor | 3 | 12.24 | | 73.46 | 14.28 | 0.0324 | 0.1944 | 2.37 |
| 21 | Survivor | 3 | 18.18 | | 75.00 | 6.81 | 0.0066 | 0.0274 | 8.00 |
| 22 | Survivor | 3 | 24.32 | | 74.32 | 1.35 | 0.0002 | 0.0007 | 52.10 |
| 27 | Survivor | 3 | 23.75 | | 66.25 | 10.00 | 0.0167 | 0.0467 | 4.66 |
| 28 | Survivor | 3 | 12.08 | | 81.31 | 6.59 | 0.0057 | 0.0385 | 7.98 |
| 29 | Survivor | 3 | 22.22 | | 70.37 | 7.40 | 0.0084 | 0.0266 | 7.12 |

**Table S1. (cont’d).**

| **Patient ID** | **Outcome** | **TT** | **L %** | **N %** | **M %** | ***BAJ*** | ***BAL*** | ***BAW*** |
| --- | --- | --- | --- | --- | --- | --- | --- | --- |
| 30 | Survivor | 3 | 55.95 | 36.90 | 7.14 | 0.0148 | 0.0098 | 4.58 |
| 31 | Survivor | 3 | 21.05 | 69.47 | 9.47 | 0.0142 | 0.0470 | 5.05 |
| 33 | Survivor | 3 | 20.87 | 67.03 | 12.08 | 0.0247 | 0.0796 | 3.51 |
| 34 | Survivor | 3 | 10.84 | 84.33 | 4.81 | 0.0028 | 0.0225 | 12.11 |
| 36 | Survivor | 3 | 7.77 | 87.77 | 4.44 | 0.0023 | 0.0265 | 12.56 |
| 37 | Survivor | 3 | 36.08 | 54.63 | 9.27 | 0.0173 | 0.0262 | 4.68 |
| 38 | Survivor | 3 | 19.10 | 73.03 | 7.86 | 0.0091 | 0.0351 | 6.57 |
| 39 | Survivor | 3 | 13.33 | 81.11 | 5.55 | 0.0040 | 0.0245 | 10.30 |
| 3 | Non-survivor | 4 | 34.83 | 61.79 | 3.37 | 0.0019 | 0.0033 | 16.71 |
| 6 | Non-survivor | 4 | 16.66 | 80.00 | 3.33 | 0.0014 | 0.0068 | 20.00 |
| 8 | Non-survivor | 4 | 49.49 | 39.39 | 11.11 | 0.0352 | 0.0280 | 2.89 |
| 10 | Survivor | 4 | 30.20 | 60.41 | 9.37 | 0.0160 | 0.0321 | 4.91 |
| 11 | Survivor | 4 | 22.22 | 60.00 | 17.77 | 0.0640 | 0.1729 | 1.87 |
| 13 | Survivor | 4 | 29.89 | 61.85 | 8.24 | 0.0119 | 0.0247 | 5.87 |
| 14 | Survivor | 4 | 22.58 | 69.89 | 7.52 | 0.0087 | 0.0271 | 6.96 |
| 15 | Survivor | 4 | 6.315 | 91.57 | 2.10 | 0.0004 | 0.0071 | 32.62 |
| 17 | Survivor | 4 | 6.38 | 80.85 | 12.76 | 0.0231 | 0.2926 | 2.11 |
| 18 | Survivor | 4 | 23.86 | 69.31 | 6.81 | 0.0071 | 0.0209 | 7.90 |
| 21 | Survivor | 4 | 13.97 | 78.49 | 7.52 | 0.0078 | 0.0438 | 6.77 |
| 22 | Survivor | 4 | 27.27 | 60.22 | 12.50 | 0.0296 | 0.0654 | 3.30 |
| 29 | Survivor | 4 | 22.72 | 63.63 | 13.63 | 0.0338 | 0.0947 | 2.91 |
| 31 | Survivor | 4 | 18.36 | 71.42 | 10.20 | 0.0162 | 0.0631 | 4.50 |
| 33 | Survivor | 4 | 36.95 | 51.08 | 11.95 | 0.0317 | 0.0439 | 3.22 |
| 36 | Survivor | 4 | 14.28 | 75.51 | 10.20 | 0.0153 | 0.0811 | 4.31 |
| 38 | Survivor | 4 | 47.61 | 41.66 | 10.71 | 0.0308 | 0.0270 | 3.17 |
| 8 | Non-survivor | 5 | 31.57 | 57.89 | 10.52 | 0.0213 | 0.0392 | 4.12 |
| 10 | Survivor | 5 | 27.84 | 64.55 | 7.59 | 0.0096 | 0.0224 | 6.67 |
| 11 | Survivor | 5 | 20.22 | 65.16 | 14.60 | 0.0383 | 0.1235 | 2.59 |
| 13 | Survivor | 5 | 23.23 | 65.65 | 11.11 | 0.0211 | 0.0597 | 3.99 |
| 14 | Survivor | 5 | 24.27 | 58.25 | 17.47 | 0.0635 | 0.1524 | 1.93 |
| 15 | Survivor | 5 | 9.18 | 82.65 | 8.16 | 0.0087 | 0.0790 | 5.36 |
| 17 | Survivor | 5 | 9.57 | 77.65 | 12.76 | 0.0240 | 0.1951 | 2.60 |
| 18 | Survivor | 5 | 24.71 | 73.03 | 2.24 | 0.0007 | 0.0020 | 29.79 |
| 22 | Survivor | 5 | 29.11 | 62.02 | 8.86 | 0.0138 | 0.0295 | 5.36 |
| 31 | Survivor | 5 | 20.00 | 68.42 | 11.57 | 0.0221 | 0.0758 | 3.74 |
| 33 | Survivor | 5 | 29.54 | 61.36 | 9.09 | 0.0148 | 0.0307 | 5.16 |
| 10 | Survivor | 6 | 29.47 | 60.00 | 10.52 | 0.0206 | 0.0420 | 4.20 |
| 13 | Survivor | 6 | 24.13 | 59.77 | 16.09 | 0.0516 | 0.1278 | 2.22 |
| 15 | Survivor | 6 | 10.10 | 83.83 | 6.06 | 0.0046 | 0.0387 | 8.64 |
| 17 | Survivor | 6 | 11.57 | 71.57 | 16.84 | 0.0476 | 0.2945 | 1.73 |
| 18 | Survivor | 6 | 25.77 | 63.91 | 10.30 | 0.0185 | 0.0459 | 4.42 |
| 22 | Survivor | 6 | 30.76 | 62.63 | 6.59 | 0.0074 | 0.0151 | 7.82 |
| 18 | Survivor | 7 | 12.12 | 76.76 | 11.11 | 0.0180 | 0.1145 | 3.60 |
| 22 | Survivor | 7 | 22.68 | 62.88 | 14.43 | 0.0387 | 0.1073 | 2.66 |

* TT: temporal test, L: lymphocyte, N: neutrophil, M: monocyte. BAJ/BAL/BAW: hypothetical and dimensionless indicators derived from L, N, and M data. >

**Table S2. Avian data.**

| **Bird ID** | **Location** | **Species** | **H %*** | **M %*** | **L %*** | ***BAW**** | **Patterns*** |
| --- | --- | --- | --- | --- | --- | --- | --- |
| MX001 | Merida Z | *Turdus* | 78.17 | 1.52 | 20.30 | 47.75 | B1 |
| MX002 | Dzoyaxche | *Turdus* | 76.80 | 1.54 | 21.64 | 46.35 | B1 |
| MX003 | Dzoyaxche | *Quiscalus* | 52.52 | 4.04 | 43.43 | 11.89 | B2 |
| MX004 | Dzoyaxche | *Turdus* | 57.44 | 4.78 | 37.76 | 10.65 | B2 |
| MX005 | Dzoyaxche | *Turdus* | 59.78 | 4.23 | 35.97 | 12.63 | B2 |
| MX006 | Dzoyaxche | *Turdus* | 58.85 | 6.77 | 34.37 | 7.26 | B2 |
| MX007 | Dzoyaxche | *Turdus* | 54.68 | 2.08 | 43.22 | 25.04 | B2 |
| MX008 | Dzoyaxche | *Turdus* | 58.11 | 3.14 | 38.74 | 17.11 | B2 |
| MX009 | Dzoyaxche | *Turdus* | 58.88 | 1.52 | 39.59 | 37.23 | B2 |
| MX010 | Merida Z | *Quiscalus* | 61.37 | 1.58 | 37.03 | 37.07 | B2 |
| MX011 | Merida Z | *Turdus* | 58.82 | 2.13 | 39.03 | 26.07 | B2 |
| MX012 | Merida Z | *Quiscalus* | 75.12 | 1.03 | 23.83 | 69.47 | B1 |
| MX013 | Merida Z | *Turdus* | 61.45 | 2.60 | 35.93 | 22.00 | B2 |
| MX014 | Dzoyaxche | *Turdus* | 75.66 | 2.64 | 21.69 | 25.49 | B2 |
| MX015 | Dzoyaxche | *Turdus* | 84.18 | 1.53 | 14.28 | 49.67 | B1 |
| MX016 | Dzoyaxche | *Turdus* | 75.77 | 1.54 | 22.68 | 45.87 | B1 |
| MX018 | Dzoyaxche | *Turdus* | 78.64 | 1.56 | 19.79 | 46.65 | B1 |
| MX019 | Merida Z | *Turdus* | 76.92 | 1.02 | 22.05 | 71.66 | B1 |
| MX020 | Dzoyaxche | *Turdus* | 84.77 | 0.50 | 14.72 | 161.43 | A |
| MX021 | Dzoyaxche | *Turdus* | 80.30 | 1.51 | 18.18 | 48.92 | B1 |
| MX022 | Dzoyaxche | *Turdus* | 81.12 | 0.51 | 18.36 | 154.70 | A |
| MX024 | Dzoyaxche | *Turdus* | 82.23 | 1.01 | 16.75 | 76.37 | B1 |
| MX025 | Dzoyaxche | *Turdus* | 82.82 | 1.01 | 16.16 | 77.17 | B1 |
| MX026 | Dzoyaxche | *Turdus* | 83.83 | 0.50 | 15.65 | 160.81 | A |
| MX027 | Dzoyaxche | *Turdus* | 82.23 | 1.01 | 16.75 | 76.37 | B1 |
| MX028 | Dzoyaxche | *Turdus* | 81.81 | 0.50 | 17.67 | 157.50 | A |
| MX029 | Dzoyaxche | *Turdus* | 80.30 | 1.51 | 18.18 | 48.92 | B1 |
| MX030 | Merida Z | *Quiscalus* | 69.07 | 0.51 | 30.41 | 131.76 | Ab |
| MX031 | Merida Z | *Turdus* | 81.12 | 1.02 | 17.85 | 75.20 | B1 |
| MX032 | Dzoyaxche | *Quiscalus* | 69.89 | 1.53 | 28.57 | 43.34 | B1 |
| MX033 | Dzoyaxche | *Turdus* | 76.56 | 1.04 | 22.39 | 70.23 | B1 |
| MX034 | Dzoyaxche | *Turdus* | 76.92 | 1.02 | 22.05 | 71.66 | B1 |
| MX035 | Dzoyaxche | *Turdus* | 66.83 | 1.02 | 32.14 | 63.48 | B1 |
| MX036 | Dzoyaxche | *Turdus* | 72.10 | 1.57 | 26.31 | 43.08 | B1 |
| MX037 | Dzoyaxche | *Turdus* | 78.57 | 1.02 | 20.40 | 73.33 | B1 |
| MX038 | Merida Z | *Turdus* | 67.34 | 1.02 | 31.63 | 63.93 | B1 |
| MX039 | Merida Z | *Turdus* | 76.41 | 1.02 | 22.56 | 71.26 | B1 |
| MX040 | Dzoyaxche | *Turdus* | 75.77 | 1.03 | 23.19 | 70.37 | B1 |
| MX042 | Dzoyaxche | *Turdus* | 76.43 | 1.57 | 21.98 | 45.42 | B1 |
| MX044 | Dzoyaxche | *Turdus* | 83.83 | 0.50 | 15.65 | 160.81 | A |
| MX046 | Dzoyaxche | *Turdus* | 71.13 | 1.03 | 27.83 | 66.53 | B1 |
| MX048 | Dzoyaxche | *Turdus* | 76.43 | 1.04 | 22.51 | 69.75 | B1 |
| MX050 | Dzoyaxche | *Turdus* | 74.73 | 1.57 | 23.68 | 44.37 | B1 |
| MX051 | Dzoyaxche | *Turdus* | 75.00 | 1.56 | 23.43 | 45.00 | B1 |
| MX052 | Merida Z | *Quiscalus* | 77.94 | 1.53 | 20.51 | 47.13 | B1 |
| MX053 | Dzoyaxche | *Quiscalus* | 76.28 | 1.03 | 22.68 | 70.78 | B1 |

**Table S2. (cont’d).**

| **Bird ID** | **Location** | **Species** | **H %*** | **M %*** | **L %*** | ***BAW**** | **Patterns*** |
| --- | --- | --- | --- | --- | --- | --- | --- |
| MX054 | Dzoyaxche | Turdus | 70.15 | 1.04 | 28.79 | 64.64 | B1 |
| MX055 | Merida Z | *Turdus* | 76.41 | 0.51 | 23.07 | 145.76 | A |
| MX057 | Dzoyaxche | *Turdus* | 61.78 | 1.57 | 36.64 | 37.71 | B2 |
| MX058 | Merida Z | *Turdus* | 69.58 | 1.03 | 29.38 | 65.21 | B1 |
| MX062 | Dzoyaxche | *Turdus* | 64.94 | 2.06 | 32.98 | 29.64 | B2 |
| MX063 | Dzoyaxche | *Turdus* | 79.89 | 0.51 | 19.58 | 151.02 | A |
| MX064 | Merida Z | *Turdus* | 69.43 | 0.51 | 30.05 | 131.72 | Ab |
| MX065 | Merida Z | *Quiscalus* | 64.28 | 2.04 | 33.67 | 29.70 | B2 |
| MX066 | Merida Z | *Turdus* | 65.60 | 2.64 | 31.74 | 22.89 | B2 |
| MX067 | Merida Z | *Turdus* | 67.91 | 1.06 | 31.01 | 61.38 | B1 |
| MX068 | Merida Z | *Turdus* | 75.38 | 1.53 | 23.07 | 45.93 | B1 |
| MX069 | Merida Z | *Turdus* | 78.94 | 1.05 | 20.00 | 71.25 | B1 |
| MX070 | Dzoyaxche | *Turdus* | 75.78 | 1.05 | 23.15 | 68.87 | B1 |
| MX072 | Dzoyaxche | *Turdus* | 68.04 | 1.03 | 30.92 | 63.87 | B1 |
| MX073 | Dzoyaxche | *Turdus* | 86.08 | 0.51 | 13.40 | 160.81 | A |
| MX076 | Merida Z | *Quiscalus* | 65.15 | 1.51 | 33.33 | 41.13 | B1 |
| MX077 | Dzoyaxche | *Quiscalus* | 76.56 | 0.52 | 22.91 | 143.73 | A |
| MX078 | Dzoyaxche | *Quiscalus* | 67.01 | 1.54 | 31.44 | 41.30 | B1 |
| MX081 | Merida Z | *Turdus* | 84.10 | 0.51 | 15.38 | 158.71 | A |
| MX082 | Merida Z | *Turdus* | 83.50 | 0.51 | 15.97 | 156.93 | A |
| MX083 | Dzoyaxche | *Turdus* | 62.17 | 2.59 | 35.23 | 22.35 | B2 |
| MX084 | Dzoyaxche | *Turdus* | 72.44 | 1.02 | 26.53 | 68.37 | B1 |
| MX087 | Dzoyaxche | *Turdus* | 69.58 | 1.54 | 28.86 | 42.71 | B1 |
| MX088 | Dzoyaxche | *Turdus* | 78.68 | 0.50 | 20.81 | 151.31 | A |
| MX089 | Dzoyaxche | *Turdus* | 68.20 | 1.53 | 30.25 | 42.18 | B1 |
| MX090 | Merida Z | *Quiscalus* | 74.24 | 1.01 | 24.74 | 70.61 | B1 |
| MX091 | Merida Z | *Quiscalus* | 77.20 | 1.03 | 21.76 | 71.11 | B1 |
| MX092 | Dzoyaxche | *Turdus* | 77.83 | 1.03 | 21.13 | 71.98 | B1 |
| MX093 | Dzoyaxche | *Turdus* | 80.51 | 0.51 | 18.97 | 152.86 | A |
| MX094 | Dzoyaxche | *Turdus* | 79.48 | 1.02 | 19.48 | 73.62 | B1 |
| MX095 | Dzoyaxche | *Turdus* | 76.26 | 1.01 | 22.72 | 72.28 | B1 |
| MX096 | Merida Z | *Turdus* | 74.09 | 0.51 | 25.38 | 140.14 | A |
| MX097 | Dzoyaxche | *Quiscalus* | 55.37 | 1.07 | 43.54 | 50.25 | Ba |
| MX099 | Dzoyaxche | *Quiscalus* | 52.30 | 1.02 | 46.66 | 49.90 | Ba |
| MX100 | Merida Z | *Quiscalus* | 73.71 | 1.03 | 25.25 | 68.69 | B1 |
| MX101 | Dzoyaxche | *Quiscalus* | 68.71 | 0.51 | 30.76 | 131.80 | Ab |
| MX102 | Dzoyaxche | *Turdus* | 77.43 | 1.02 | 21.53 | 72.06 | B1 |
| MX103 | Merida Z | *Quiscalus* | 75.39 | 0.52 | 24.08 | 140.93 | A |
| MX105 | Merida Z | *Quiscalus* | 80.61 | 1.02 | 18.36 | 74.84 | B1 |
| MX106 | Merida Z | *Quiscalus* | 80.85 | 1.06 | 18.08 | 71.77 | B1 |
| MX110 | Dzoyaxche | *Turdus* | 80.21 | 1.09 | 18.68 | 68.94 | B1 |
| MX112 | Dzoyaxche | *Quiscalus* | 77.55 | 1.02 | 21.42 | 72.54 | B1 |
| MX115 | Merida Z | *Turdus* | 78.35 | 1.03 | 20.61 | 72.38 | B1 |
| MX120 | Dzoyaxche | *Quiscalus* | 70.10 | 2.06 | 27.83 | 31.65 | B2 |

| MX122 | Dzoyaxche | Turdus | 73.71 | 1.03 | 25.25 | 68.69 | B1 |
| --- | --- | --- | --- | --- | --- | --- | --- |
| MX123 | Dzoyaxche | Turdus | 73.46 | 1.02 | 25.51 | 69.23 | B1 |
| MX125 | Dzoyaxche | *Turdus* | 72.44 | 1.02 | 26.53 | 68.37 | B1 |
| MX126 | Dzoyaxche | *Turdus* | 78.78 | 1.01 | 20.20 | 74.28 | B1 |

**Table S2. (cont’d).**

* H−L: heterophil, monocyte, lymphocyte; *BAW*: a dimensionless number derived from blood data; Patterns: data subsets consisting with (i) no inflammation (Ba), (ii) early inflammation (A, B1), (iii) late inflammation (B2), and (iv) other (Ab).
