# Supplementary material for: Assessing the Dynamics and Complexity of Disease Pathogenicity Using 4-Dimensional Immunological Data
Source: Front Immunol. 2019 Jun 12;10:1258. doi: 10.3389/fimmu.2019.01258 (PMC6582751; doi:10.3389/fimmu.2019.01258)
Supplement: Supplementary file 3 [file Presentation_1.PPT]

## Slide 1
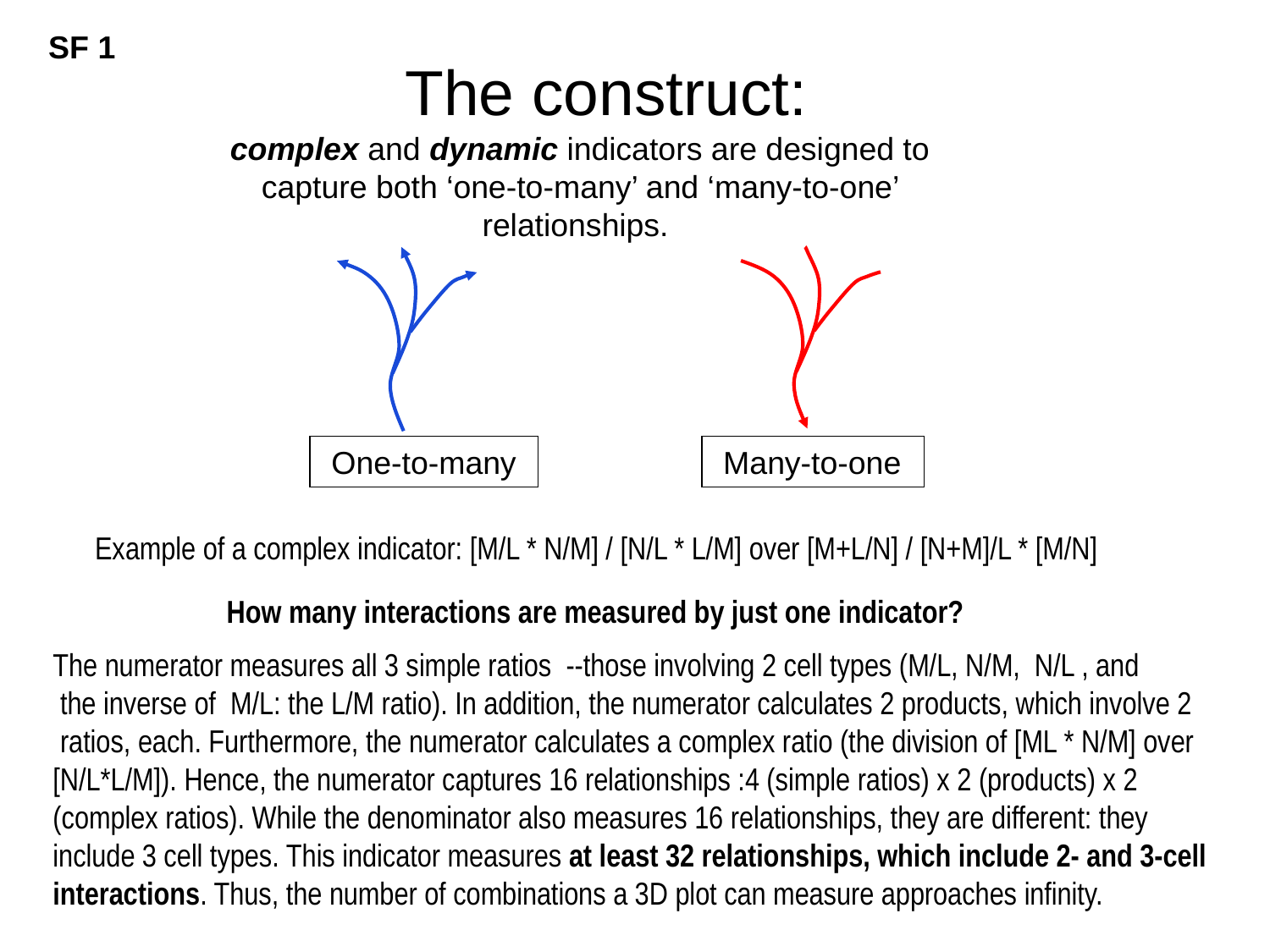

SF 1
 The construct:
complex and dynamic indicators are designed to capture both ‘one-to-many’ and ‘many-to-one’ relationships.
 One-to-many
 Many-to-one
Example of a complex indicator: [M/L * N/M] / [N/L * L/M] over [M+L/N] / [N+M]/L * [M/N]
How many interactions are measured by just one indicator?
The numerator measures all 3 simple ratios --those involving 2 cell types (M/L, N/M, N/L , and
 the inverse of M/L: the L/M ratio). In addition, the numerator calculates 2 products, which involve 2
 ratios, each. Furthermore, the numerator calculates a complex ratio (the division of [ML * N/M] over
[N/L*L/M]). Hence, the numerator captures 16 relationships :4 (simple ratios) x 2 (products) x 2
(complex ratios). While the denominator also measures 16 relationships, they are different: they
include 3 cell types. This indicator measures at least 32 relationships, which include 2- and 3-cell
interactions. Thus, the number of combinations a 3D plot can measure approaches infinity.

## Slide 2
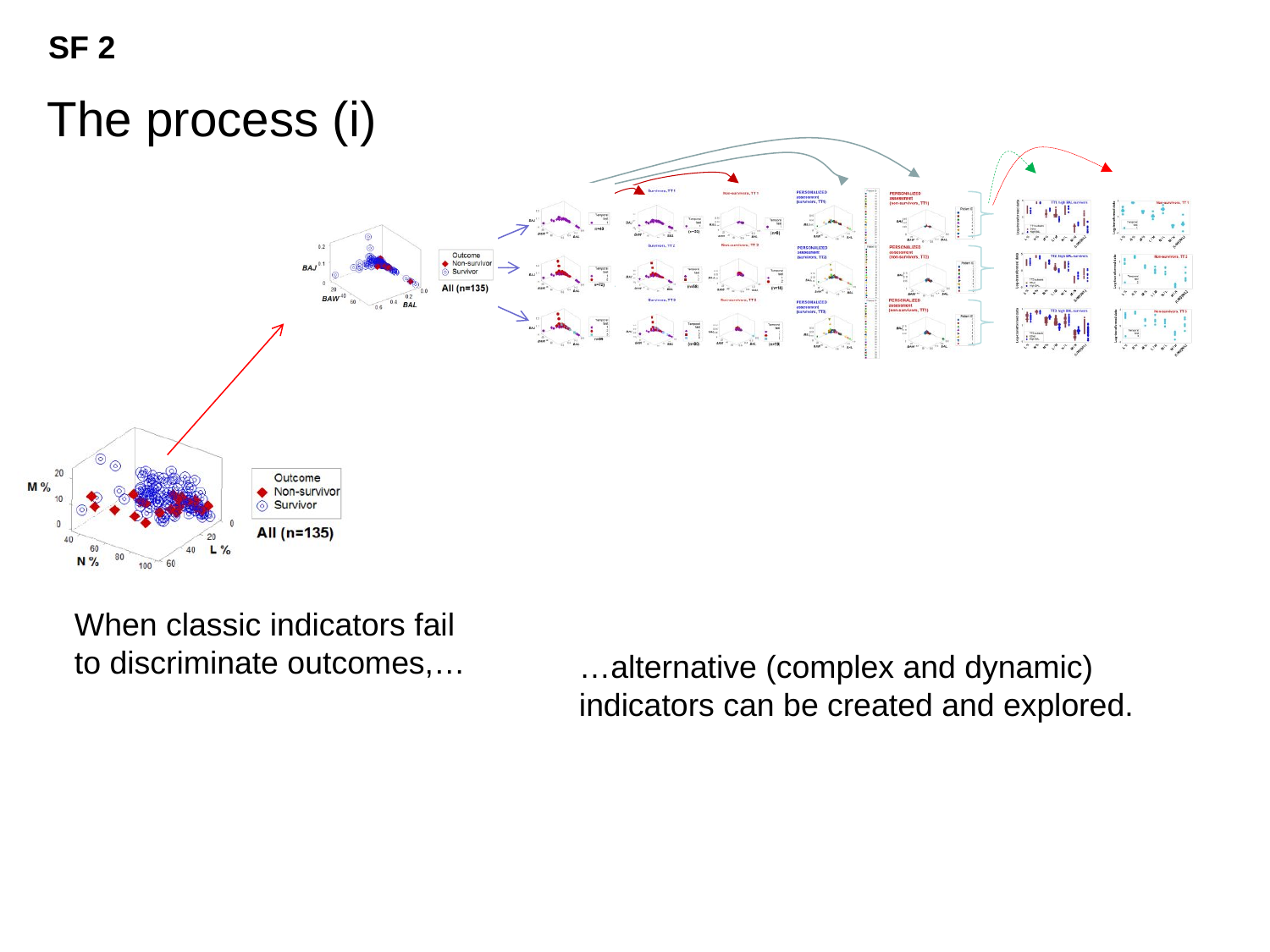

SF 2
The process (i)
When classic indicators fail to discriminate outcomes,…
…alternative (complex and dynamic) indicators can be created and explored.

## Slide 3
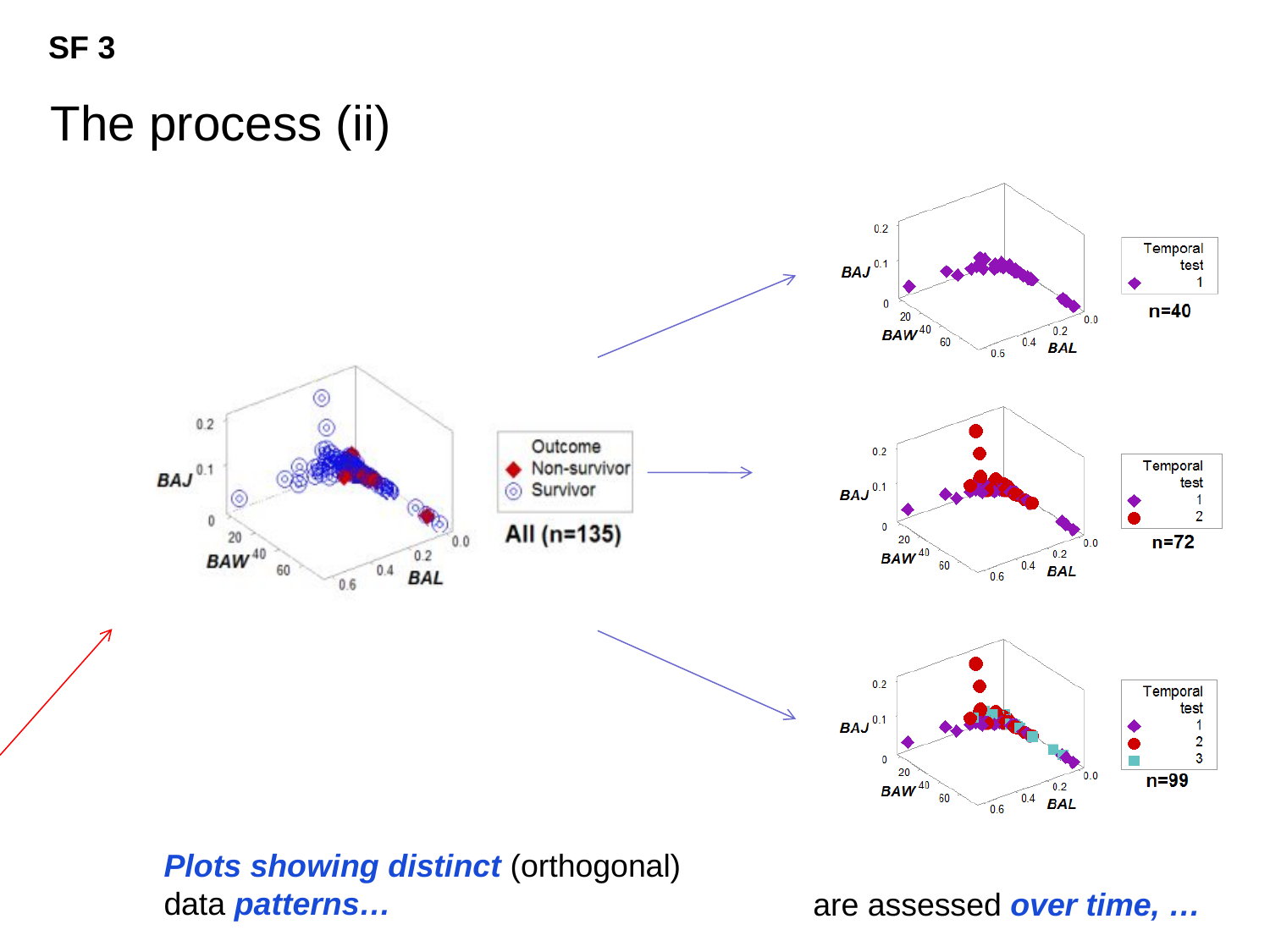

SF 3
The process (ii)
Plots showing distinct (orthogonal) data patterns…
 are assessed over time, …

## Slide 4
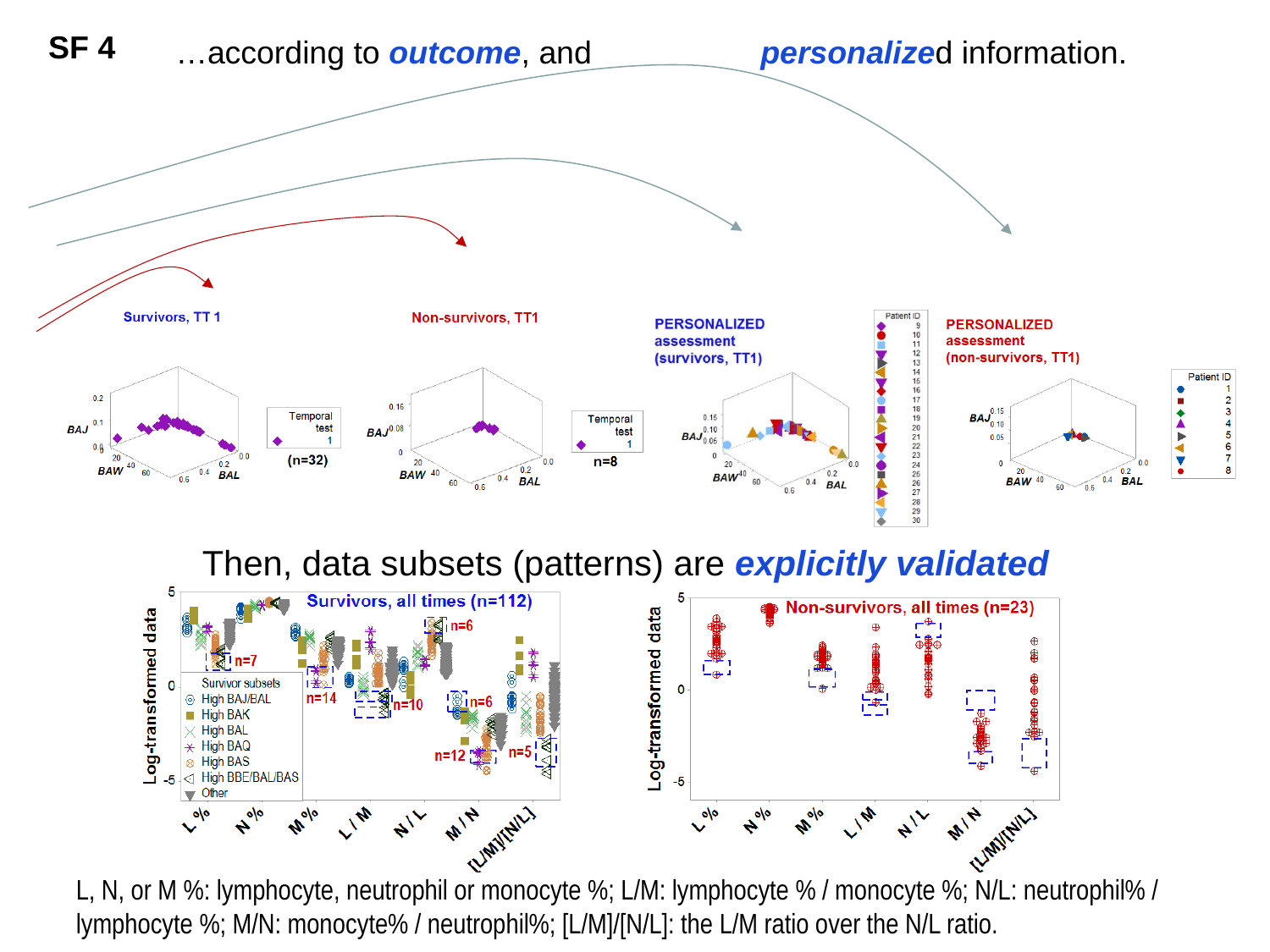

SF 4
…according to outcome, and personalized information.
Then, data subsets (patterns) are explicitly validated
L, N, or M %: lymphocyte, neutrophil or monocyte %; L/M: lymphocyte % / monocyte %; N/L: neutrophil% / lymphocyte %; M/N: monocyte% / neutrophil%; [L/M]/[N/L]: the L/M ratio over the N/L ratio.

## Slide 5
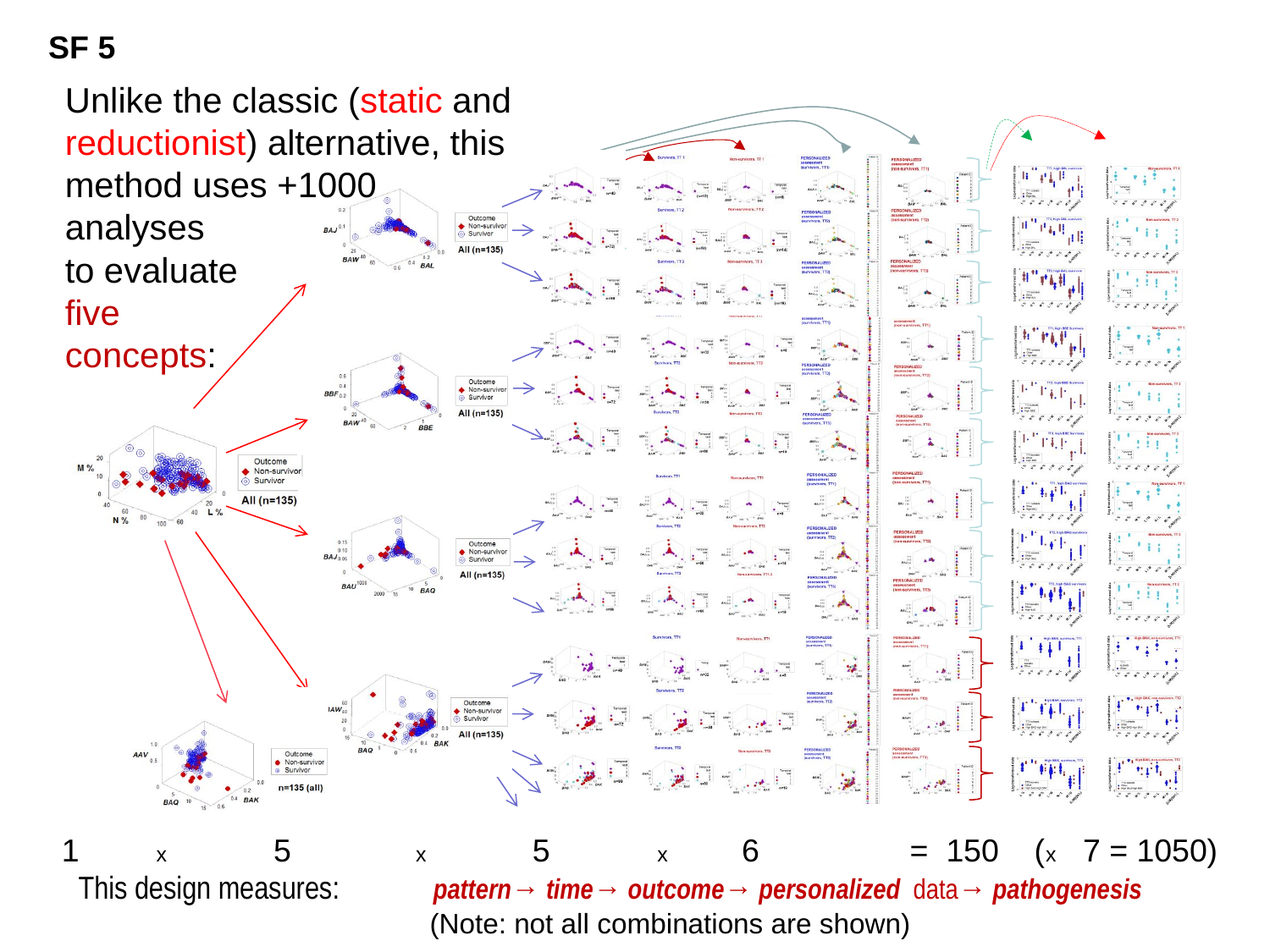

SF 5
Unlike the classic (static and reductionist) alternative, this method uses +1000
analyses
to evaluate
five
concepts:
 1 x 5 x 5 x 6 = 150 (x 7 = 1050)
 This design measures: pattern→ time→ outcome→ personalized data→ pathogenesis
 (Note: not all combinations are shown)

## Slide 6
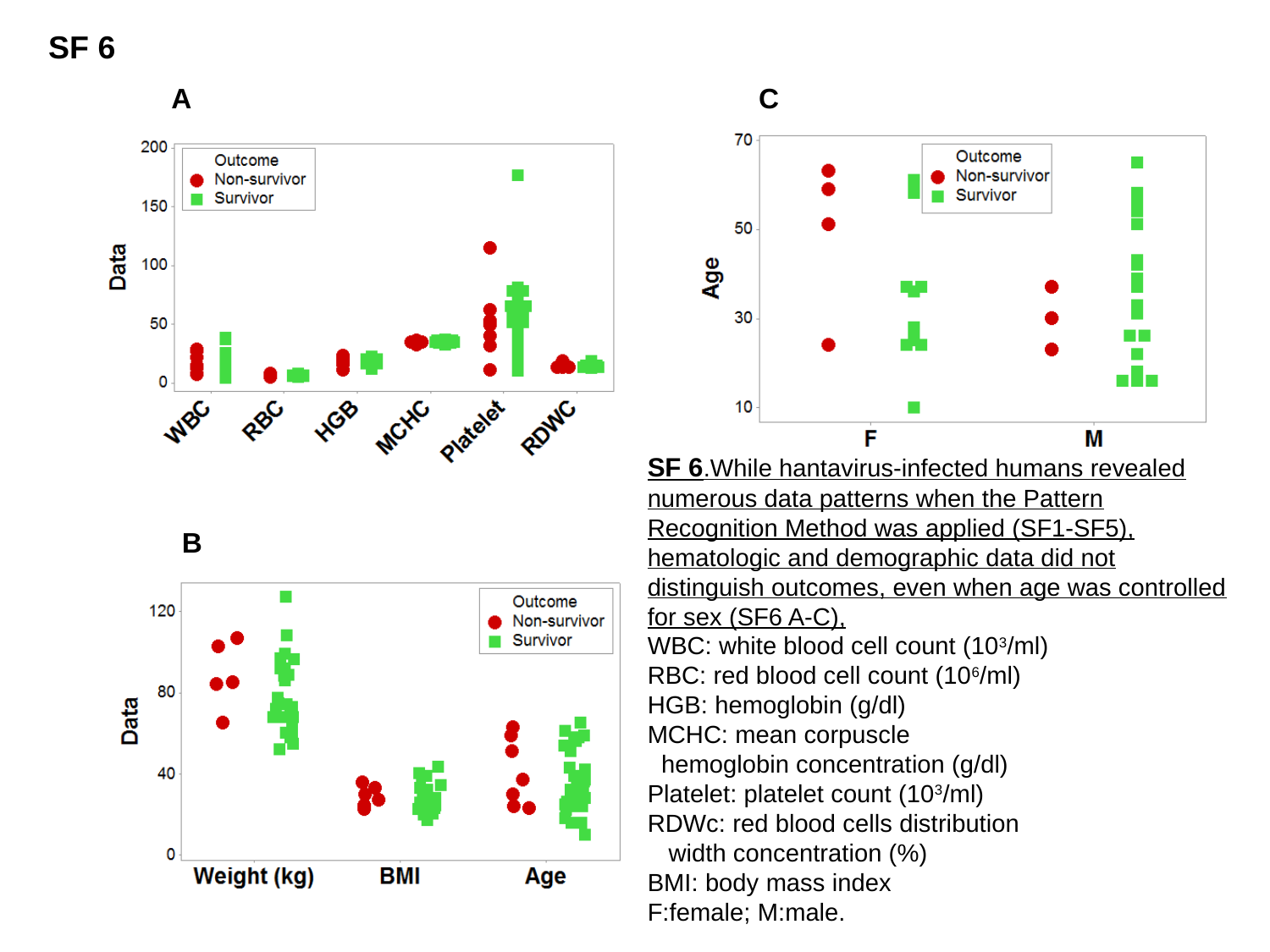

SF 6
A
C
SF 6.While hantavirus-infected humans revealed numerous data patterns when the Pattern Recognition Method was applied (SF1-SF5), hematologic and demographic data did not distinguish outcomes, even when age was controlled for sex (SF6 A-C),
WBC: white blood cell count (103/ml)
RBC: red blood cell count (106/ml)
HGB: hemoglobin (g/dl)
MCHC: mean corpuscle
 hemoglobin concentration (g/dl)
Platelet: platelet count (103/ml)
RDWc: red blood cells distribution
 width concentration (%)
BMI: body mass index
F:female; M:male.
B
